# The changing landscape of expanded access to investigational drugs for patients with unmet medical needs: ethical implications

**DOI:** 10.1186/s40545-017-0100-3

**Published:** 2017-02-21

**Authors:** Eline M. Bunnik, Nikkie Aarts, Suzanne van de Vathorst

**Affiliations:** 1 Department of Medical Ethics and Philosophy of Medicine, Erasmus MC, University Medical Centre Rotterdam, Wytemaweg 80, 3015 CN Rotterdam, The Netherlands; 2 Amsterdam Medical Centre (AMC), Department of General Practice, Meibergdreef 9, 1105 AZ Amsterdam, The Netherlands

**Keywords:** Expanded access, Compassionate use, Investigational drugs, Ethical issues

## Abstract

When patients are told that standard medical treatment options have been exhausted, their treating physicians may start looking for promising new drugs that are not yet approved, and still under investigation. Some patients can be included in clinical trials, but others cannot. It is not widely known that these patients might still be eligible for trying investigational drugs, in a therapeutic context. Worldwide, public and private parties are seeking to change this by informing patients and physicians about opportunities for expanded access and/or by facilitating its processes. When expanded access becomes available to larger groups of patients, ethical issues gain prominence, including informed consent, funding issues, disparities in access, and potential adverse effects on clinical drug development. Physicians, patients and policy-makers should not shift the responsibility to address these issues to pharmaceutical companies, but work together to resolve them.

Many countries have regulatory programs in place for expanded access, also known as ‘compassionate use’ or ‘named-patient’ programs. Expanded access programs are usually restricted to patients suffering from serious or life-threatening diseases who have run out of standard treatment options and cannot enroll in clinical trials, e.g. because they do not meet the inclusion criteria. Requests for expanded access are made by treating physicians and evaluated by drug regulatory authorities. The pharmaceutical company usually supplies the drug at no cost. In many countries, the uptake of expanded access is low [1, 2]. This is likely the result of both knowledge deficits and financial issues. Firstly, physicians are not always aware of the possibility to prescribe drugs that are not yet approved for marketing [3]. When they do, they may be held back by unfamiliarity with the regulatory process, administrative burdens, safety and/or liability concerns [4]. Patients and patient organizations, likewise, seem to know little about expanded access programs [5]. Secondly, in the absence of any (economic) incentives to supply investigational drugs, few companies are willing or able to provide expanded access ‘out of compassion’.

Around the world, public and private initiatives are currently trying to increase the availability of expanded access beyond the highly selective group of patients who are now able to use it. In the US in recent years, the Right-to-Try movement has raised public awareness of existing options for expanded access. Also, it has led to the introduction of Right-to-Try legislation in over 30 American states, giving terminally ill patients the right to use unapproved drugs directly, without approval from the Food and Drug Administration (FDA). Despite broad public and political support, Right-to-Try laws have been criticized for offering no more than ‘false hope’ to patients with unmet medical needs [6]. The laws do not require pharmaceutical companies to release the unapproved drugs, leaving patients without claim rights. In effect, these laws seem to have led to little improvement in making unapproved drugs more accessible for patients [6]. Some patients and patient advocacy groups are using a different approach: Kids v. Cancer, for example, has developed a ‘Compassionate Use Navigator’, a clearinghouse of information about application processes for expanded access, which also offers personal assistance for the pediatric oncology community. A similar initiative to inform patients can be found, for instance, on the website of EURORDIS, an advocacy group for rare diseases in Europe. Also, the FDA has significantly improved its application process for expanded access to reduce the administrative burdens on physicians: it should now take no more than 45 min to finalize a request [3]. Furthermore, commercial initiatives are stepping in to increase accessibility of expanded access for patients. For instance, the company myTomorrows has developed a global online platform to inform patients and physicians worldwide about local opportunities and regulations for expanded access, and distributes a range of investigational drugs through its own expanded access programs. Idis, a contract research organization, runs so-called Managed Access programs on behalf of pharmaceutical companies.

All these efforts – albeit different in many ways – share one aim: to make expanded access a *real option* for a much wider range of patients with unmet medical needs, either through informing patients about expanded access or by facilitating its processes. But, as a greater uptake of unapproved drugs comes unlocked, ethical issues associated with expanded access become more pressing. For instance, the ethical requirement of informed consent may be difficult to meet: physicians must ensure that patients understand the ‘experimental’ nature of the treatment, the lack of complete safety and efficacy data, and possible risks. Even properly informed patients may still be biased by therapeutic misconception [7]: an over-optimistic interpretation of potential benefits and an underestimation of potential harms. Some patients, however, will be well aware of their limited chances at medical benefit, and nevertheless willing to try. Furthermore, it is feared, expanded access may thwart clinical trial enrolment, for patients will be less willing to participate in randomized controlled trials when they can access investigational drugs directly [8]. Thus, expanded access for individual patients runs counter to the goal of clinical drug development: the marketing authorization of safe and effective drugs for the benefit of entire patient populations. This concern, however, can be addressed by the requirement that patients only qualify for expanded access when they cannot be included in clinical trials, which is already in place in many countries.

Most importantly, efforts to inform patients about expanded access may be in vain as long as major obstacles are not removed, including knowledge gaps among physicians. It is imperative that physicians are educated about possibilities for expanded access and its regulatory processes. Information and education may also help address physicians’ liability concerns. In addition, solutions should be sought for the general reluctance among pharmaceutical companies to supply investigational drugs outside the context of clinical trials. Commercial initiatives like myTomorrows and Idis Managed Access can assist companies with the expertise and infrastructure required for offering investigational drugs through expanded access programs. Moreover, in some countries, companies are allowed to charge, either freely or ‘direct costs’ only [9] for unapproved drugs, which may incentivize them to open up to expanded access. But the problem of funding in expanded access runs deeper: health insurers in many countries are commonly unwilling to reimburse the costs of unapproved drugs. Whereas some patients will be able to amass the required sums, out of pocket or through fund-raising campaigns [5], others will not, which gives rise to ethical concerns related to equal access.

Some of these challenges can be overcome through the development of consistent policies for expanded access. As of yet, companies have not been transparent about the conditions under which they will agree to provide expanded access. The recently adopted *21st Century Cures Act* in the US requires that companies develop policies for expanded access and make these policies publicly known on their websites [10]. Other countries should follow suit, for it is important that patients and physicians know what to expect from expanded access. There is no consensus yet, however, on what adequate policies for expanded access look like, or which criteria should be used to determine whether or not patients are eligible for expanded access. The responsibility to devise the contours of such policies should not be shifted onto pharmaceutical companies, but taken on by a variety of stakeholders, including physicians, patients, policy-makers and payers. Together, they must address the fundamental question whether or not there is a place for expanded access in their healthcare systems, and if yes, how this place should be set up to allow for the responsible use of investigational drugs in patients who have run out of standard treatment options and cannot participate in clinical trials.

## Conclusions

Efforts to facilitate expanded access will only give patients ‘false hope’ as long as current practical barriers and ethical issues are not tackled. These include knowledge gaps, financial constraints, regulatory processes, health disparities, and threats to clinical drug development. Over the next few years, patients, physicians, policy-makers and pharmaceutical companies must work together to develop policies and systems to ensure equitable, safe and well-informed expanded access for patients with unmet medical needs.
